# The Spread of Scientific Information: Insights from the Web Usage Statistics in PLoS Article-Level Metrics

**DOI:** 10.1371/journal.pone.0019917

**Published:** 2011-05-16

**Authors:** Koon-Kiu Yan, Mark Gerstein

**Affiliations:** 1 Department of Molecular Biophysics and Biochemistry, Yale University, New Haven, Connecticut, United States of America; 2 Program in Computational Biology and Bioinformatics, Yale University, New Haven, Connecticut, United States of America; 3 Department of Computer Science, Yale University, New Haven, Connecticut, United States of America; Center for Complex Networks and Systems research, Indiana University at Bloomington, United States of America

## Abstract

The presence of web-based communities is a distinctive signature of Web 2.0. The web-based feature means that information propagation within each community is highly facilitated, promoting complex collective dynamics in view of information exchange. In this work, we focus on a community of scientists and study, in particular, how the awareness of a scientific paper is spread. Our work is based on the web usage statistics obtained from the PLoS Article Level Metrics dataset compiled by PLoS. The cumulative number of HTML views was found to follow a long tail distribution which is reasonably well-fitted by a lognormal one. We modeled the diffusion of information by a random multiplicative process, and thus extracted the rates of information spread at different stages after the publication of a paper. We found that the spread of information displays two distinct decay regimes: a rapid downfall in the first month after publication, and a gradual power law decay afterwards. We identified these two regimes with two distinct driving processes: a short-term behavior driven by the fame of a paper, and a long-term behavior consistent with citation statistics. The patterns of information spread were found to be remarkably similar in data from different journals, but there are intrinsic differences for different types of web usage (HTML views and PDF downloads versus XML). These similarities and differences shed light on the theoretical understanding of different complex systems, as well as a better design of the corresponding web applications that is of high potential marketing impact.

## Introduction

In the era of Web 2.0, individuals are tightly connected in the virtual worlds and form various online communities. Recently, the propagation of information in these online communities has gained much attention [1], partly because of the massive popularity of online social networking sites and their potential marketing impact. Information propagation is a complex dynamical process rooted in the interactions between huge numbers of heterogeneous individuals. As online communities by their nature capture a complete record of their members, they offer unprecedented opportunities to study emergent properties of human behaviors. Generally speaking, a community is characterized by a common interest. For example, users from the website CNET (http://www.cnet.com/) form a community that are primary interested in information about gadgets; scientists following a certain set of scientific journals comprise another community in which the awareness of scientific papers is being spread. Exploring the propagation of information in different communities sheds light on the intrinsic differences between different types of information, and it is interesting to question whether different communities share any universal behavior. So far most studies have focused on popular communities of general users like digg.com, myspace and Flickr, and little work has been done on more specific communities in which the number of users is smaller, and the users tend to be more homogeneous. In this work, we look at the signatures of scientific information by focusing on how the awareness of scientific papers spread within specific communities of scientists.

Over the last two decades, the WWW has revolutionized scientific research, in particular by speeding up the rate of the spread of information. Nowadays, once a paper is electronically published on a journal website, the information can propagate rapidly in the community, partially due to various scientific blogs and folksonomy websites like CiteULike and Connotea. The spread of a paper will then be reflected at the level of web usage statistics, in particular, the number of HTML views, i.e. the WWW traffic of the webpage corresponding to the paper. In this work, we regard readers of the 6 PLoS journals (PLoS Biology, PLoS Computational Biology, PLoS Genetics, PLoS Medicine, PLoS One and PLoS Pathogens) as a community of scientists. As an estimation of the size of the community, there were over 4000 papers published in 2008 and the total HTML views numbered over 7 million. We quantitatively examine the propagation process by studying the monthly web usage statistics of individual papers reported in the PLoS Article-Level Metrics (ALM) dataset. The dataset contains the number of HTML views; the number of PDF and XML downloads of more than 13000 papers published from 2003 to 2009 on a monthly basis since their publication. Compiled by PLoS, the ALM dataset (http://www.plos.org/cms/node/485) also includes various statistics such as the number of citations, blog coverage and social bookmarking. These statistics are designed to provide a more thorough measure of the impact of a paper.

## Results

### Correlation between various statistics in the PLoS Article-Level Metrics

**Figure 1:**
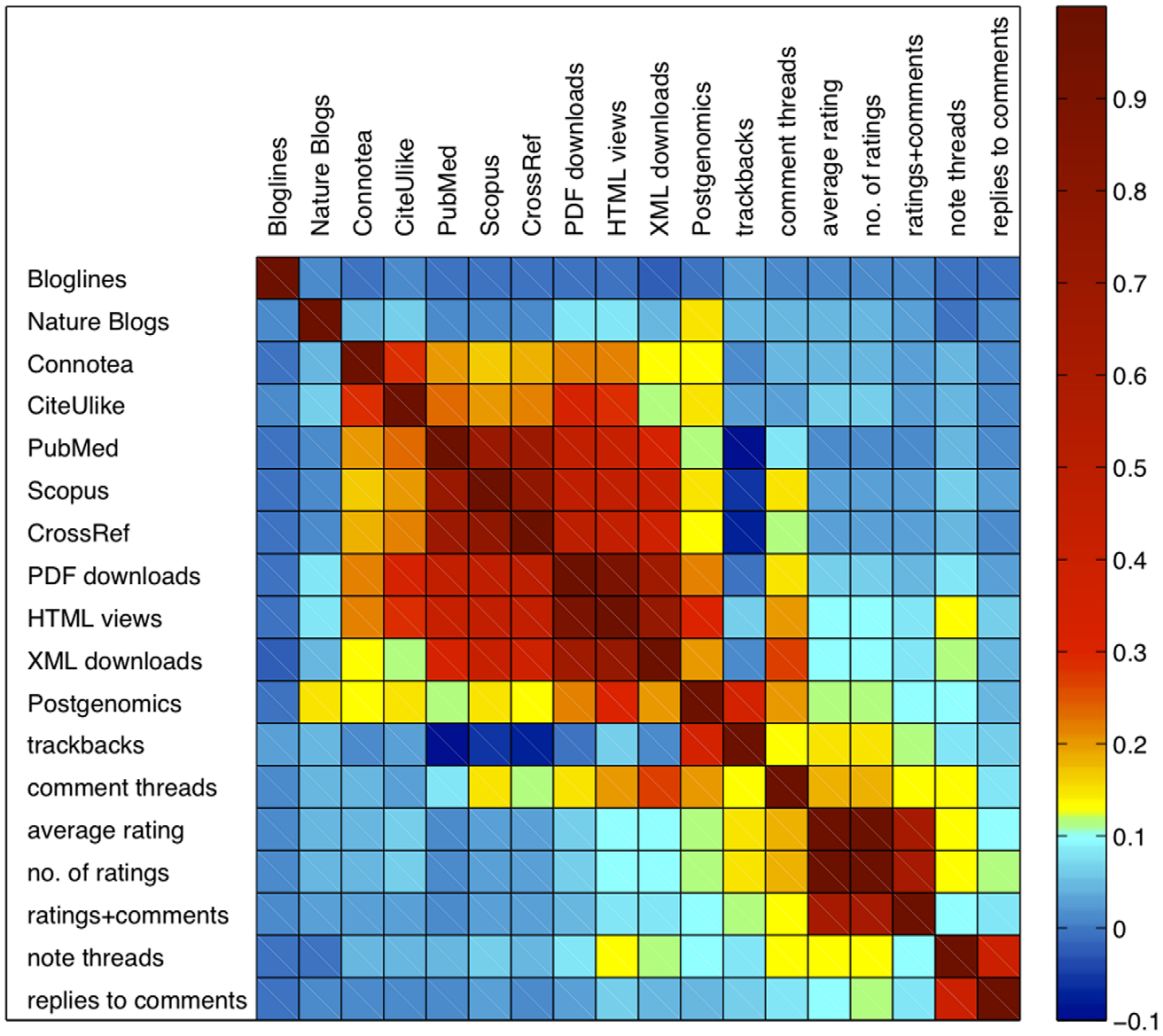
The Spearman correlation between various Article Level Metrics compiled by PLoS. The full meaning of the labels are: note threads (number of notes users put on an article), replies to comments (number of replies to comment threads of an article), rating+comments (number of users who leave a rating as well as a comment to an article), no. of ratings (how many times an article has been rated), average rating (the average rating an article received), comment threads (number of comment threads users put on an article), trackbacks (the number of trackbacks that have been made to this article by external sites), Bloglines, Nature Blogs and Postgenomics (the number of times an article have been blogged by the respective sites, Connotea and CiteUlike (the counts of how many bookmarks have been made to an article by users of these social bookmarking sites), CrossRef, PubMed and Scopus (the counts of how many citations are recorded in these databases), HTML views, PDF downloads and XML downloads (the counts of HTML views, PDF and XML downloads for each article). The article access metrics, the citation metrics and the social bookmarking metrics form a broad cluster.

To develop a better intuition regarding the PLoS Article-Level Metrics, we examined the correlation pattern between various statistics. Figure 1 shows the Spearman correlation matrix of 18 different metrics, including the article usage statistics (HTML views, PDF downloads, XML downloads), citation statistics (PubMed, CrossRef, Scopus), blog coverage (Bloglines, Nature Blogs, Postgenomics), social bookmarking (CiteUlike, Connotea) and various online ratings employed in the PLoS website. As shown in Figure 1, the article access metrics, the citation metrics and the social bookmarking metrics broadly form a cluster, signified by relatively high correlation coefficients among them. It is interesting to point out that the number of citations is best correlated with the access statistics (with average spearman correlation r = 0.44, the highest correlation with the number of PDF downloads (r = 0.48)), and then the number of bookmarking (average spearman correlation r = 0.2). Among the article access statistics, the number of PDF downloads strongly correlates with the number of HTML views (r = 0.91, P = 0), and these article access statistics generally agree with social bookmarking metrics (Connotea and CiteUlike) and blog coverage metric (Postgenomics), suggesting media coverage or spread of knowledge between individuals might contribute to the access statistics of articles, or vice versa.

### Decay in the number of web accesses

**Figure 2:**
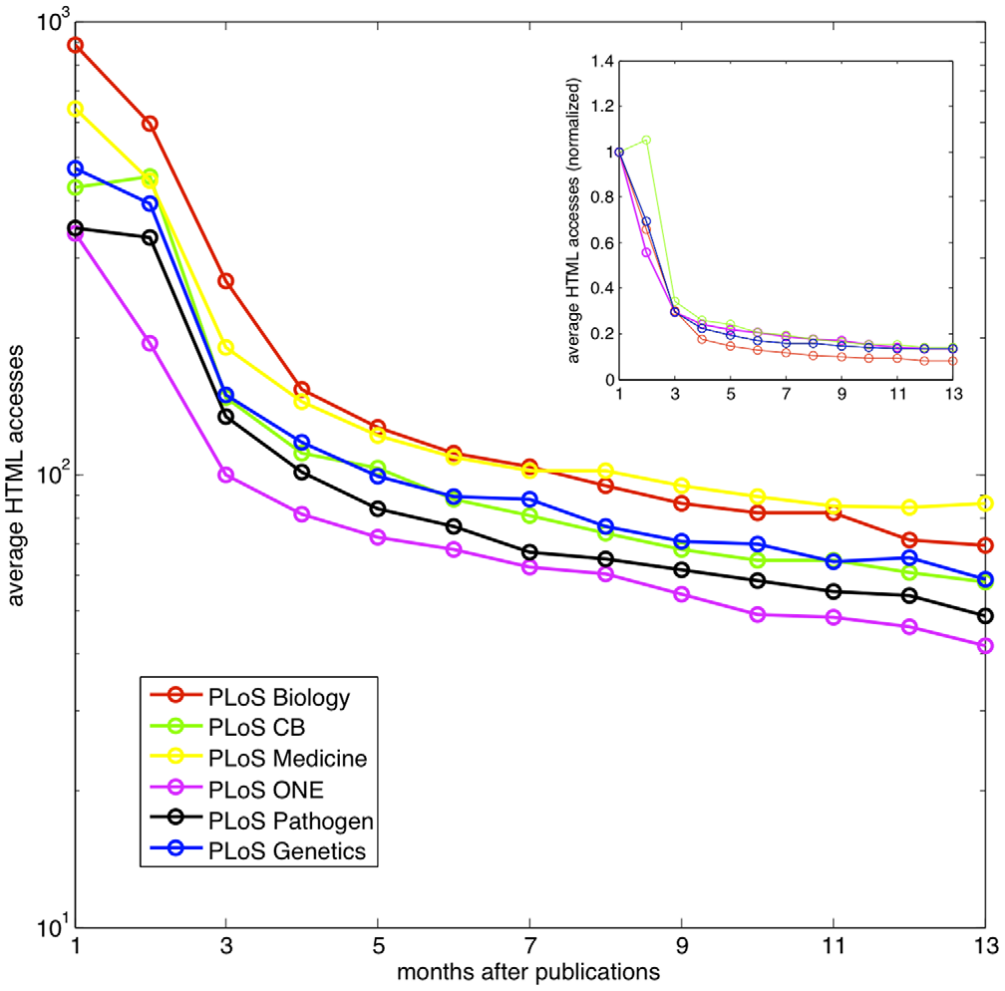
The average number of HTML views of articles in six PLoS journals. We study the access statistics of 7000 publications that have been published for at least one year. These publications belong to 6 different PLoS journals: PLoS Biology (1177), PLoS Computational Biology (688), PLoS Genetics (723), PLoS Medicine (1300), PLoS One (2796) and PLoS Pathogen (543). In average, the number of HTML views of an old paper is lower. The decay process is much faster from the first month to the second month after publication, compared to the subsequent period. The inset shows the average accesses of different journals normalized by the corresponding values of the first month. Note that the patterns are remarkably similar for the six different journals.

We next move our focus to the empirical observation of information propagation using a time series of web accesses. As described above, the number of PDF downloads and the number of HTML views are strongly correlated, and we thus use the HTML views as a proxy to measure information propagation. We considered 7000 papers that have been published for at least one year, and counted the number of HTML views they received at different time points after publication. These papers were published in one of the six PLoS journals: PLoS Biology, PLoS Computational Biology, PLoS Genetics, PLoS Medicine, PLoS One and PLoS Pathogens. Figure 2 shows the decrease of the average number of views. As expected, on average, the older a paper is, the less attention it receives. In particular, from the first month to the second month, the decay is rapid, while later on the decay goes slower. Moreover, it is interesting to point out that such decay patterns are remarkably similar for the six different PLoS journals listed (see the inset of Figure 2), including ones for broad audiences like PLoS Biology, and ones for more specialized readers like PLoS Computional Biology.

**Figure 3:**
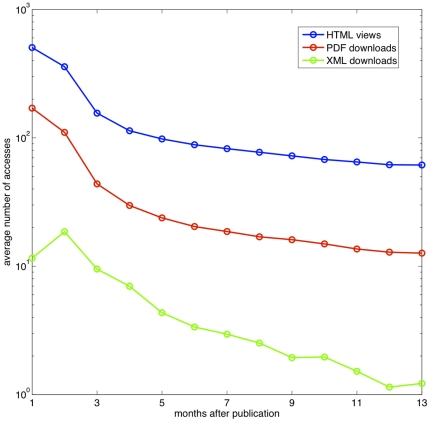
The decay patterns of three types of web accesses: HTML views, PDF downloads, and XML downloads. The decay pattern of PDF downloads are consistent with the pattern of HTML views. Both profiles possess the same two phases of decay. The profile of XML downloads does not share the same characteristics.

While the number of HTML views better reflects the knowledge of the existence of a paper, we repeated the decay pattern analysis for the number of PDF downloads, which might arguably measure the number of times a paper is read, and also the number of XML downloads. Figure 3 shows the decay patterns for all three types of web accesses. Being consistent with the high correlation observed in Figure 1, the decay pattern of PDF downloads resemble the pattern of HTML views in the sense both of them possess the two phases of decay. Nevertheless, as shown in Figure 3, the decay of XML downloads does not share the same characteristics.

### The long tail distribution of cumulative number of HTML views

**Figure 4:**
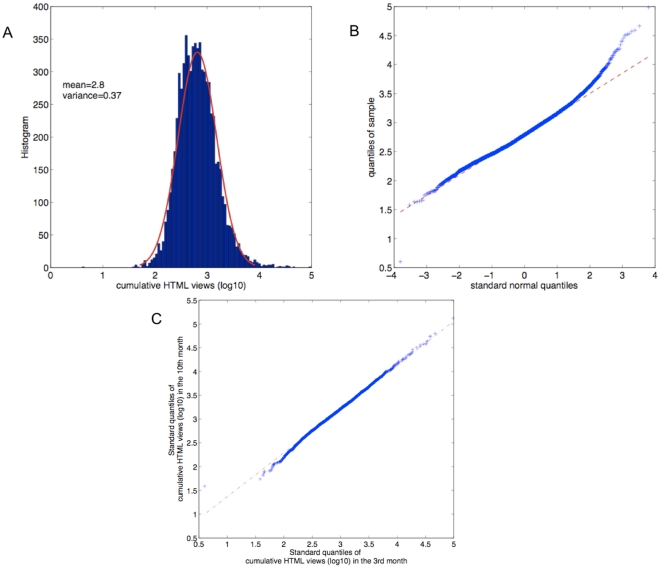
The number of cumulative HTML views follows a long tail distribution, reasonable well fitted by a lognormal distribution. A. The cumulative number of HTML views of 7000 papers at the 3^rd^ month after publication is fitted using the maximum likelihood method by a lognormal distribution, with the mean and variance of the logarithmic values as shown. B. The normal Q-Q plot of the logarithm of the HTML views shown in panel A. Apart from a slightly longer tail, the plot is close to a straight line, meaning that the majority of the data is well explained by a lognormal distribution. C. The Q-Q plot between the cumulative accesses of the same set of 7000 papers at the 3^rd^ month and at the 10^th^ month after publication. The plot is very close to a straight line, suggesting the cumulative HTML views at different time points follow the same distribution up to certain shift and scaling factors.

In addition to the average number of HTML accesses as shown in Figure 2, we studied how the number of accesses of individual papers are distributed. For the 7000 papers that have been published for at least one year, we examined the cumulative number of HTML views at a time 3 months after publication. As shown in Figure 4A, the logarithm of the numbers is reasonably well fitted by a normal distribution using the maximum likelihood method, suggesting that the number of HTML views of the 7000 papers follows a lognormal distribution. We then looked at the normal Q-Q plot of the logarithm of the HTML views. Apart from a slightly longer tail, the plot is close to a straight line (Figure 4B), meaning that the majority of the data are well explained by a lognormal distribution.

While the lognormal distribution is a reasonable approximation to the distribution of cumulative HTML views at a certain instant, we ask whether this case is true for any time point. As the lifespan of papers increases, the cumulative views of all individual papers increase monotonically. As shown in Figure 4C, the Q-Q plots between the cumulative accesses at two different months are all very close to straight lines. This result suggests that the cumulative HTML views at different times follow the same distribution up to certain shift and scaling factors.

### A stochastic model of information propagation

**Figure 5:**
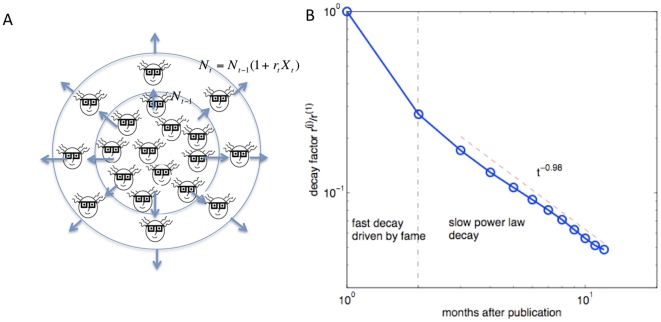
A stochastic model of information diffusion. A. After a scientist has accessed a paper, he/she might spread information from the paper to his friends, colleagues or students. The information would then be further spread via a cascade of social interactions. The cumulative number of accesses at time t is pictured by the number of scientists enclosed in the concentric circles. Mathematically, 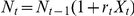, where X_t_ s are positive, independently and identically distributed random variables with finite mean 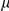 and variance 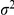, and r_t_ is a modulating factor (see main text). B. Modulating factors decay with respect to time. The value at the jth month, r^(j)^, is normalized by the value at the first month r^(1)^. The decay of modulating factors is divided into two regimes: a rapid drop from the first month to the second month, and a low power law decay afterward. The power law regime is best fitted by the function 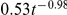, with 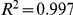. The residuals for the points in the second regime are in order of 10^−3^. The residual of the first data point, compared to the fitted curve, is 0.48. The point is significantly deviated from the power law regime.

It is well known that lognormal distributions can be generated by the so-called random multiplicative processes. A simple stochastic model, which was recently used by Wu and Huberman in a study of the voting statistics in digg.com [2], can be easily applied in our scenario to examine information propagation in a scientific community. There are two basic assumptions in this model: 1.) After a scientist has accessed a paper (and hopefully read it as well), he/she might spread the information of the paper to his friends, colleagues or students. The information would then be further spread via a cascade of social interactions. 2.) Independent from the intrinsic properties of the paper, say relevance and quality, the chance of someone passing on the information in an old paper is less than that of passing on a new paper. Suppose N_t_ is the cumulative number of HTML views at time t. The dynamical process is mathematically written as 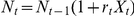, where X_i_ are positive, independent and identically distributed random variables with finite mean 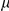 and variance 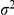 (Figure 5A). The mean 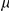 can be interpreted as, on average, the fraction of scientists who would spread the information in each step of the cascade. The additional parameter r_t_, is defined to moderate the average rate of spread of information at time t. As the time series are given in the resolution of month, r_t_ is a piecewise constant function such that 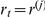 if t is at the jth month after publication.

The simple model is able to explain the observed lognormal distributions. When time steps are small, X_t_ is small and therefore we write 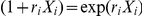. The cumulative accesses of a paper at time t can be written as 
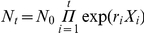
, where N_0_ is the size of initial sources. Taking the logarithm of both sides, we have 
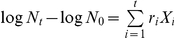
. The righthand side converges to a normal distribution. As N_0_ is comparably small, the cumulative accesses of a paper could be viewed as a random variable drawn from a lognormal distribution. Furthermore, the average value 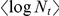 at the k months after publishing is proportional to the sum of the modulating factors, given by 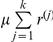.

By fitting the data to the stochastic model, we further extracted the parameters r^(j)^, which are proportional to the rates of information propagation at different stages. As shown in Figure 5B, there are two phases of decay: the rate drops rapidly from the first month to the second month after publication, and then a slow decay which follows a power law with exponent −0.98. Indeed, the second regime is well fitted by the power law (R^2^ = 0.997), the initial rapid decay is significantly deviated from this power law (see caption for details).

## Discussion

We have empirically studied the online access statistics in the PLoS ALM dataset. We proposed to use the number of HTML views of a paper to quantify by how far the paper has percolated in the scientific community, and explained the time series of HTML views using a simple stochastic model. We found that the rate of information spread decreases as a function of time after publication, and there are two decay regimes: a rapid drop from the first month to the second month after publication, and a slow power law decay afterward (Figure 5B). The power law decay is not unexpected. The pattern is consistent with the scenario of receiving citations. While researchers tend to have an exponentially decaying memory regarding the papers they cite (i.e. a researcher is exponentially more likely to cite a new paper than an old paper) [3] [4], for a single publication, the number of citations it received as a function of time decays in a power law fashion [5]. In particular, as reported in Ref. [5], the power law exponent is −0.94, which is remarkably close to the decay exponent we observed in accesses. Of particular interest is the extremely high access number in the first month and the corresponding rapid decay. We refer to this behavior as an effect of fame, which is probably exaggerated in modern days due to immediate online media coverage. What is the underlying mechanism of fame? Due to the low temporal resolution of the time series (in a monthly basis), the PLoS ALM data is not able to provide enough insights. However, with a higher temporal resolution, several studies have shed light to the question in the framework of relaxation dynamics. These studies focus on systems like the number of views of videos in YouTube [6], the sales of books and music in Amazon [7], and the amount of donation in response to the tsunami at Dec, 2004 [8]. For instance, with time series in a resolution in a daily basis, Ref. [6] identified a class of videos whose viewing involves nontrivial herding behavior analogously to what we referred to as fame. Nevertheless, in terms of downloads of publications, it is still worthwhile to explore at the level of individual paper, the relationship between the fame received at the beginning and the later impact of a paper, the number of downloads later on, and even the number of citations.

We have emphasized that a lognormal distribution is a reasonable approximation of the empirical data. Nevertheless, as shown in Figure 3, the tail of the distribution is not perfectly fitted by a lognormal distribution, suggesting that the current model cannot fully capture the dynamics of publications that receive extremely high attention. These extremely high downloads could be the results of mechanisms such as the Matthew effect (richer get richer) [9]. It is important to mention that the current formalism is a mean-field model, in which details such as the inhomogeneity between different research fields, or the sub-communities structure among scientists are not captured. With the availability of many more social networks among research communities, future work could be done on more specific propagation channels, for instance the social networks built on folksonomy resources such as CiteULike, or in an almost real-time fashion: the twitter network [10]. To explore the inhomogeneity between different research fields, we repeated the analysis of Figure 2 for papers in different topics (see Methods). As shown in Figure S1, the accesses of different groups of papers decrease in a similar fashion.

The emergence of lognormal distribution via random multiplicative processes has been studied for a long time [11], and appears in a wide range of applications. Examples include the MacArthur model for species abundance [12] and the Black-Scholes Model in finance [13]. More recently, it has been used to for the description of popularity patterns in many contexts such as Internet traffic of websites [14] [15], proportional elections [16] and citation statistics [17] [18] [19]. In particular, Huberman et.al. used a random multiplicative process to model the number of votes a story received in digg.com [2]. Ref. [2] introduced the concept of novelty and explained the voting statistics in terms of the decay of novelty. Although the idea of novelty is similar to what we refer to as information in this study, the pattern of two decay regimes is not observed in the voting statistics. This is because, unlike scientific literature, news articles appearing in digg.com do not have long-term followers, and fame thus dominates the voting process.

We have questioned whether the decay patterns are consistent among three types of web usage statistics: the number of HTML views and the number of PDF and XML downloads. Interesting enough, the number of XML downloads is not consistent with the other two. The fact that XML downloads behave differently from the usage of both HTML and PDF is also presented in the correlation map shown in Figure 1. This is probably because XML downloads are performed by machines, and thus the decay profile follows a different characteristic time scale. In fact, unlike the usage of HTML and PDF, the number of XML downloads does not follow a lognormal distribution. This is not entirely surprising as machines visit websites regularly without talking to each other; the underlying mechanism is not a random multiplicative process.

Over the last decades, electronic publications have revolutionized our ways of publishing, leading to many interesting new questions as well as methods in scientometrics. For instance, the relationship between the number of citations acquired by an article to the number of downloads or accesses [20] [21], as well as the effects of new practices such as open access publishing [22], have been explored. Recently, the sequences of access of articles (clickstream) have been used to generate a visual map of science [23]. We believe that the era of Web 2.0 will bring further questions and challenges to scientometrics or bibliometrics. For instance, metrics like the PLoS Article-Level metrics will become more and more popular, as there will be many different ways for a paper to be exposed to the community, and thus the impact of a paper would not be able to be merely quantified by the number of citations. More importantly, data such as the web access statistics enable us to further identify and quantify the collective effects of scientists. The fame and the spread of information described in this study is only one of many interesting collective phenomena.

## Methods

The PLoS Article-Level Metric (ALM) dataset was downloaded from the PLoS website (http://www.plos.org/cms/node/485) in August 2009. The dataset contains information about 13828 papers published from 2003 to 2009 in 8 PLoS journals: PLoS Biology, PLoS Computational Biology, PLoS Genetics, PLoS Medicine, PLoS One, PLoS Pathogens, PLoS Neglected Tropical Diseases and PLoS Clinical Trials. For each article, one or several topic areas are assigned. We focused on the web access statistics, in which the number of HTML views, the number of PDF and XML downloads of each article are given on a monthly basis after their publication.

## Supporting Information

Figure S1 **The average number of HTML views of articles in different topics.** The 7000 papers that have been published for more than a year are classified into different topics by PLoS ALM dataset. We plot the median number of HTML accesses against the time of publications for several selected topics. The number of papers in each of the selected topics are: mathematics (446), science policy (324), physics (110), public health and epidemiology (1729), molecular bio (1742), neuroscience (1805). Note that a paper could be classified into more than one topic. The trends of different topics are consistent to one another.
(TIFF)
