# Supplementary figures and images for: The Spread of Scientific Information: Insights from the Web Usage Statistics in PLoS Article-Level Metrics
Source: PLoS One. 2011 May 16;6(5):e19917. doi: 10.1371/journal.pone.0019917 (PMC3095621; doi:10.1371/journal.pone.0019917)

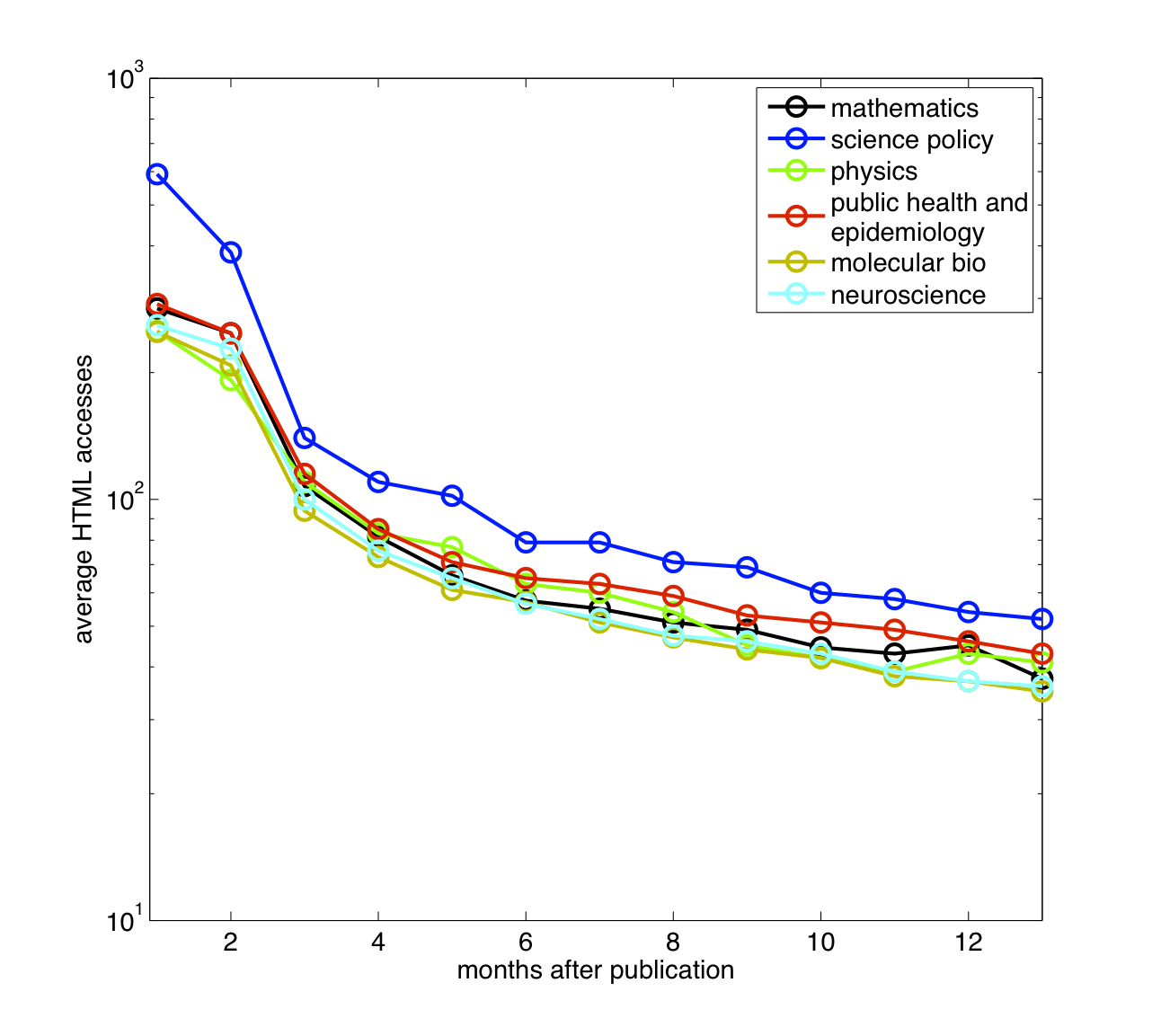

Supplement: Figure S1 — The average number of HTML views of articles in different topics. The 7000 papers that have been published for more than a year are classified into different topics by PLoS ALM dataset. We plot the median number of HTML accesses against the time of publications for several selected topics. The number of papers in each of the selected topics are: mathematics (446), science policy (324), physics (110), public health and epidemiology (1729), molecular bio (1742), neuroscience (1805). Note that a paper could be classified into more than one topic. The trends of different topics are consistent to one another. (TIFF) [file pone.0019917.s001.tif]
